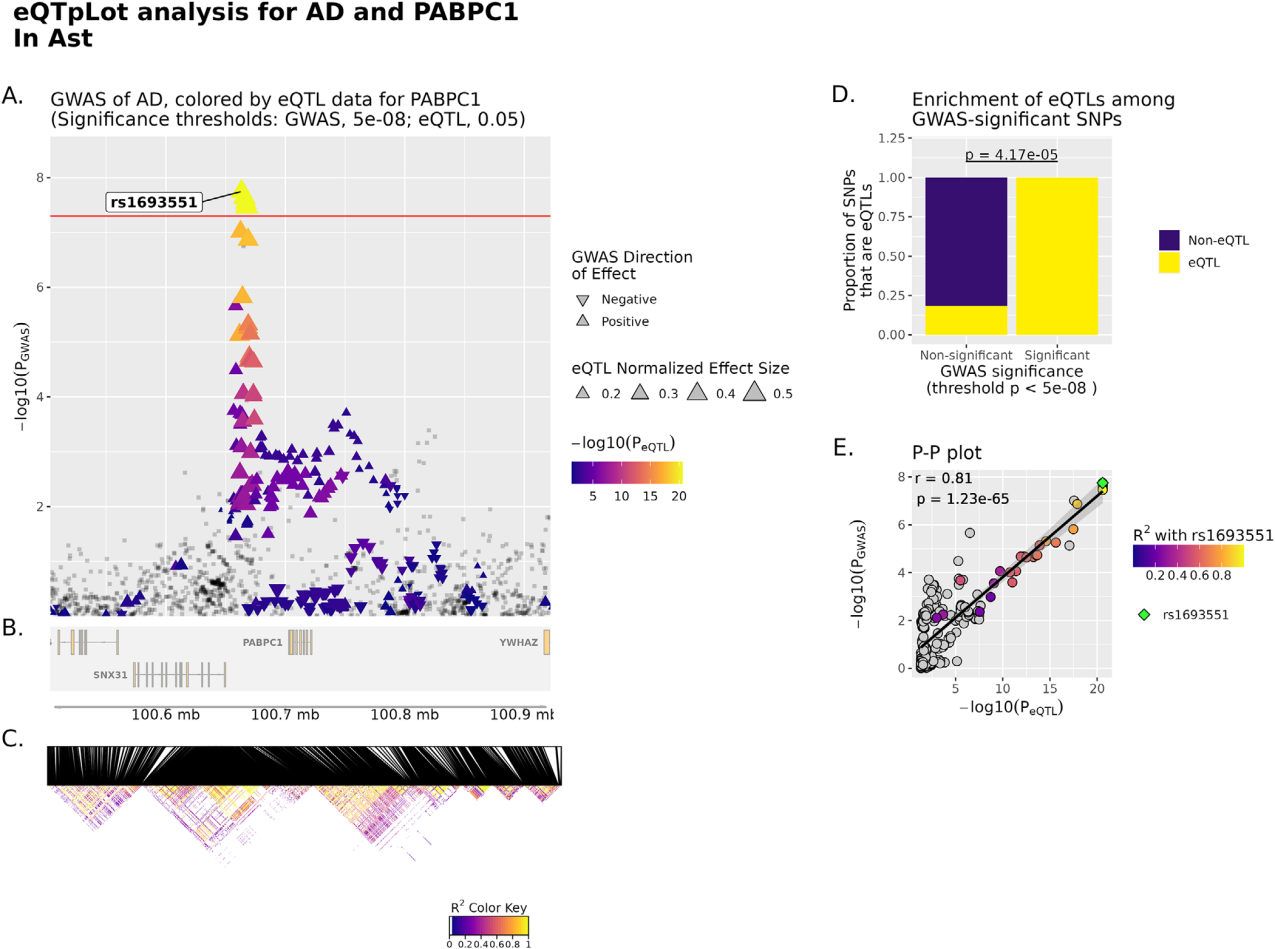# Integration of GWAS summary statistics with cell type‐specific eQTLs prioritizes potential causal genes for Alzheimer’s disease

**DOI:** 10.1002/alz.095701

**Published:** 2025-01-09

**Authors:** Shiwei Liu, Yen‐Ning Huang, Tamina Park, Soumilee Chaudhuri, Min Young Cho, Thea Jacobson Rosewood, David A. Bennett, Andrew J. Saykin, Kwangsik Nho

**Affiliations:** ^1^ Indiana University School of Medicine, Indianapolis, IN USA; ^2^ Rush Alzheimer’s Disease Center, Rush University Medical Center, Chicago, IL USA

## Abstract

**Background:**

Analyzing disease‐linked genetic variants via expression quantitative trait loci (eQTLs) is crucial for identifying disease‐causing genes. Previous research prioritized genes by integrating Genome‐Wide Association Study (GWAS) results with tissue‐level eQTLs. Recent studies explored brain cell type‐specific eQTLs, but they lack a systematic analysis across various AD GWAS datasets, nor did they compare effects between tissue and cell type levels or across different cell type‐specific eQTL datasets. Here, we integrated brain cell type‐specific eQTL datasets with AD GWAS datasets to identify potential causal genes at the cell type level.

**Method:**

To prioritize disease‐causing genes, we used summary data‐based Mendelian Randomization (SMR) and Bayesian colocalization (COLOC) methods to integrate the AD GWAS summary statistics with cell type‐specific eQTLs in human brain. We utilized five latest AD GWAS datasets and a cell type‐specific eQTL dataset comprising 424 participants of the Religious Orders Study (ROS) and Rush Memory and Aging Project (MAP) cohort. We replicated our analysis using a cell type‐specific eQTL dataset of 192 participants from Bryois et al., 2021. For comparison, we utilized a previous tissue‐level metabrain eQTL dataset from a meta‐analysis of 14 datasets. Furthermore, we visualized the colocalization of novel candidate causal genes using eQTpLot.

**Result:**

We identified 17 cell type‐specific candidate causal genes using the ROSMAP eQTL dataset. Our results showed that the largest number of candidate causal genes are identified in microglia, followed by astrocytes, oligodendrocytes, excitatory neurons, inhibitory neurons, and oligodendrocyte progenitor cells (OPCs). Four candidate causal genes were common across different cell types. Interestingly, *JAZF1*, detected as a candidate causal gene affected by the same leading variant in both microglia and OPCs, showed a congruous (same direction) colocalized SNP effect on the gene expression level and AD in OPCs, but an incongruous (opposite direction) colocalized SNP effect in microglia. After comparing our results with previously known prioritized causal genes, we identified *PABPC1* in astrocyte as a novel potential causal gene.

**Conclusion:**

We systematically prioritized AD candidate causal genes based on cell type‐specific molecular evidence. The integrative approach enhances our understanding of molecular mechanisms of AD‐related genetic variants and facilitates the interpretation of AD GWAS results.